# *N*,*N*′-Bis[(*E*)-2-Benzyl­idenepropylidene]ethane-1,2-diamine

**DOI:** 10.1107/S1600536808025919

**Published:** 2008-08-16

**Authors:** Aliakbar Dehno Khalaji, Seik Weng Ng

**Affiliations:** a Department of Science, Gorgan University of Agricultural Sciences and Natural Resources, Gorgan 49189-43464, Iran; b Department of Chemistry, University of Malaya, 50603 Kuala Lumpur, Malaysia

## Abstract

The two independent mol­ecules in the asymmetric unit of the title Schiff base, C_22_H_24_N_2_, lie across centers of inversion. The C=N double bonds are in a *trans* configuration.

## Related literature

There are many examples of similar Schiff bases in the current (2008) Cambrige Structural Database; for example, see: Khalaji *et al.* (2007 ▶). For the structure of bis­[(*E*)-3-phenyl­propen-1-al]-1,2-diimino­ethane, see: Khalaji & Weil (2007 ▶).
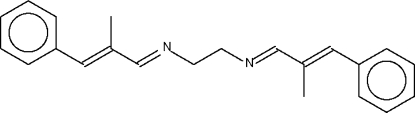


## Experimental

### 

#### Crystal data

C_22_H_24_N_2_
*M* *_r_* = 316.43
Triclinic, 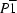

*a* = 9.524 (2) Å
*b* = 9.576 (2) Å
*c* = 10.202 (2) Å
α = 88.160 (3)°
β = 76.865 (2)°
γ = 78.651 (3)°
*V* = 888.3 (3) Å^3^
*Z* = 2
Mo *K*α radiation
μ = 0.07 mm^−1^
*T* = 100 (2) K
0.24 × 0.12 × 0.08 mm

#### Data collection

Bruker SMART APEX diffractometer
Absorption correction: none
4114 measured reflections
3015 independent reflections
2186 reflections with *I* > 2σ(*I*)
*R*_int_ = 0.023

#### Refinement

*R*[*F* ^2^ > 2σ(*F* ^2^)] = 0.069
*wR*(*F* ^2^) = 0.228
*S* = 1.13
3015 reflections
219 parameters
H-atom parameters constrained
Δρ_max_ = 0.34 e Å^−3^
Δρ_min_ = −0.30 e Å^−3^

### 

Data collection: *APEX2* (Bruker, 2007 ▶); cell refinement: *SAINT* (Bruker, 2007 ▶); data reduction: *SAINT*; program(s) used to solve structure: *SHELXS97* (Sheldrick, 2008 ▶); program(s) used to refine structure: *SHELXL97* (Sheldrick, 2008 ▶); molecular graphics: *X-SEED* (Barbour, 2001 ▶); software used to prepare material for publication: *publCIF* (Westrip, 2008 ▶).

## Supplementary Material

Crystal structure: contains datablocks global, I. DOI: 10.1107/S1600536808025919/xu2448sup1.cif

Structure factors: contains datablocks I. DOI: 10.1107/S1600536808025919/xu2448Isup2.hkl

Additional supplementary materials:  crystallographic information; 3D view; checkCIF report

## Figures and Tables

**Fig. 1.**
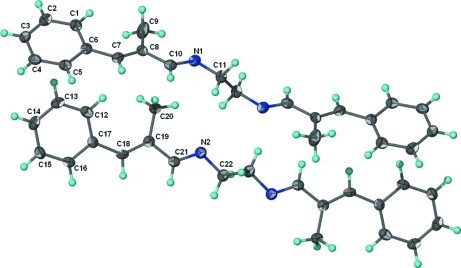
Thermal ellipsoid plot (Barbour, 2001) of the two independent molecules of the title compound; probability ellipsoids are set at the 70% level, and H atoms are drawn as spheres of arbitrary radius. The two molecules lie about centers of inversion.

## supplementary crystallographic information

### Comment

(type here to add)

### Experimental

Ethylenediamine (1 mmol, 60 mg) and α-methylcinnamaldehyde (2 mmol, 292 mg) were dissolved in methanol (10 ml) to give a colorless solution. Slow evaporation of the solvent yielded colorless crystals in about 85% yield.

### Refinement

Carbon-bound H-atoms were placed in calculated positions (C—H 0.95 to 0.98 Å) and were included in the refinement in the riding model approximation, with *U*(H) fixed at 1.2 to 1.5*U*(C).

### Crystal data

**Table:**

C_22_H_24_N_2_	*Z* = 2
*M**_r_* = 316.43	*F*_000_ = 340
Triclinic, *P*1	*D*_x_ = 1.183 Mg m^−^^3^
Hall symbol: -P 1	Mo *K*α radiation λ = 0.71073 Å
*a* = 9.524 (2) Å	Cell parameters from 1031 reflections
*b* = 9.576 (2) Å	θ = 3.2–28.0º
*c* = 10.202 (2) Å	µ = 0.07 mm^−^^1^
α = 88.160 (3)º	*T* = 100 (2) K
β = 76.865 (2)º	Block, colorless
γ = 78.651 (3)º	0.24 × 0.12 × 0.08 mm
*V* = 888.3 (3) Å^3^	

### Data collection

**Table:**

Bruker SMART APEX diffractometer	2186 reflections with *I* > 2σ(*I*)
Radiation source: fine-focus sealed tube	*R*_int_ = 0.023
Monochromator: graphite	θ_max_ = 25.0º
*T* = 100(2) K	θ_min_ = 2.1º
ω scans	*h* = −11→11
Absorption correction: None	*k* = −11→7
4114 measured reflections	*l* = −12→12
3015 independent reflections	

### Refinement

**Table:**

Refinement on *F*^2^	Secondary atom site location: difference Fourier map
Least-squares matrix: full	Hydrogen site location: inferred from neighbouring sites
*R*[*F*^2^ > 2σ(*F*^2^)] = 0.069	H-atom parameters constrained
*wR*(*F*^2^) = 0.228	*w* = 1/[σ^2^(*F*_o_^2^) + (0.0966*P*)^2^ + 1.385*P*] where *P* = (*F*_o_^2^ + 2*F*_c_^2^)/3
*S* = 1.13	(Δ/σ)_max_ = 0.001
3015 reflections	Δρ_max_ = 0.34 e Å^−^^3^
219 parameters	Δρ_min_ = −0.30 e Å^−^^3^
Primary atom site location: structure-invariant direct methods	Extinction correction: none

### Fractional atomic coordinates and isotropic or equivalent isotropic displacement parameters (Å^2^)

**Table:**

	*x*	*y*	*z*	*U*_iso_*/*U*_eq_	
N1	0.3982 (3)	1.1256 (3)	0.1398 (3)	0.0221 (7)	
N2	0.3642 (3)	0.5970 (3)	0.1443 (3)	0.0220 (7)	
C1	0.1158 (4)	1.3426 (4)	0.6498 (4)	0.0242 (8)	
H1	0.0674	1.3700	0.5785	0.029*	
C2	0.0423 (4)	1.3777 (4)	0.7810 (4)	0.0268 (9)	
H2	−0.0567	1.4279	0.7993	0.032*	
C3	0.1114 (4)	1.3405 (4)	0.8862 (4)	0.0238 (8)	
H3	0.0597	1.3646	0.9762	0.029*	
C4	0.2550 (4)	1.2685 (4)	0.8602 (4)	0.0238 (8)	
H4	0.3026	1.2428	0.9322	0.029*	
C5	0.3297 (4)	1.2335 (4)	0.7291 (3)	0.0218 (8)	
H5	0.4296	1.1860	0.7118	0.026*	
C6	0.2613 (4)	1.2668 (4)	0.6210 (3)	0.0209 (8)	
C7	0.3478 (4)	1.2169 (4)	0.4872 (3)	0.0211 (8)	
H7	0.4501	1.1871	0.4819	0.025*	
C8	0.3067 (4)	1.2062 (4)	0.3699 (3)	0.0198 (8)	
C9	0.1543 (4)	1.2473 (5)	0.3469 (4)	0.0358 (10)	
H9A	0.0849	1.2137	0.4217	0.054*	
H9B	0.1502	1.2039	0.2625	0.054*	
H9C	0.1279	1.3512	0.3415	0.054*	
C10	0.4227 (4)	1.1443 (4)	0.2552 (3)	0.0209 (8)	
H10	0.5204	1.1169	0.2671	0.025*	
C11	0.5216 (4)	1.0601 (4)	0.0345 (3)	0.0224 (8)	
H11A	0.6070	1.0211	0.0738	0.027*	
H11B	0.5500	1.1325	−0.0326	0.027*	
C12	0.1872 (4)	0.8841 (4)	0.6385 (4)	0.0234 (8)	
H12	0.1826	0.9391	0.5599	0.028*	
C13	0.1387 (4)	0.9502 (4)	0.7643 (4)	0.0235 (8)	
H13	0.1002	1.0497	0.7709	0.028*	
C14	0.1461 (4)	0.8727 (4)	0.8798 (4)	0.0252 (8)	
H14	0.1117	0.9184	0.9656	0.030*	
C15	0.2040 (4)	0.7277 (4)	0.8698 (4)	0.0238 (8)	
H15	0.2112	0.6740	0.9488	0.029*	
C16	0.2510 (4)	0.6617 (4)	0.7450 (4)	0.0224 (8)	
H16	0.2901	0.5622	0.7394	0.027*	
C17	0.2427 (3)	0.7372 (4)	0.6261 (3)	0.0188 (8)	
C18	0.2977 (4)	0.6565 (4)	0.4993 (4)	0.0210 (8)	
H18	0.3495	0.5626	0.5085	0.025*	
C19	0.2885 (4)	0.6906 (4)	0.3718 (3)	0.0201 (8)	
C20	0.2131 (4)	0.8296 (4)	0.3247 (4)	0.0260 (9)	
H20A	0.1151	0.8581	0.3833	0.039*	
H20B	0.2041	0.8184	0.2320	0.039*	
H20C	0.2711	0.9028	0.3282	0.039*	
C21	0.3610 (4)	0.5804 (4)	0.2695 (3)	0.0207 (8)	
H21	0.4084	0.4915	0.2977	0.025*	
C22	0.4398 (4)	0.4776 (4)	0.0556 (3)	0.0245 (8)	
H22A	0.3687	0.4421	0.0145	0.029*	
H22B	0.4845	0.3993	0.1079	0.029*	

### Atomic displacement parameters (Å^2^)

**Table:**

	*U*^11^	*U*^22^	*U*^33^	*U*^12^	*U*^13^	*U*^23^
N1	0.0217 (15)	0.0204 (16)	0.0237 (17)	−0.0038 (12)	−0.0043 (13)	−0.0017 (12)
N2	0.0225 (15)	0.0213 (16)	0.0224 (17)	−0.0058 (12)	−0.0041 (12)	0.0005 (12)
C1	0.0238 (18)	0.026 (2)	0.0222 (19)	−0.0025 (15)	−0.0076 (15)	0.0024 (15)
C2	0.0238 (19)	0.027 (2)	0.028 (2)	−0.0021 (16)	−0.0043 (16)	−0.0027 (16)
C3	0.0293 (19)	0.0191 (19)	0.0221 (19)	−0.0049 (15)	−0.0035 (15)	−0.0022 (15)
C4	0.030 (2)	0.0163 (18)	0.027 (2)	−0.0048 (15)	−0.0112 (16)	0.0010 (15)
C5	0.0228 (18)	0.0181 (18)	0.026 (2)	−0.0040 (15)	−0.0085 (15)	0.0012 (15)
C6	0.0238 (18)	0.0178 (18)	0.0226 (19)	−0.0077 (14)	−0.0054 (15)	0.0012 (14)
C7	0.0198 (17)	0.0186 (18)	0.0239 (19)	−0.0034 (14)	−0.0031 (15)	−0.0003 (15)
C8	0.0233 (18)	0.0190 (18)	0.0191 (18)	−0.0075 (15)	−0.0062 (15)	0.0033 (14)
C9	0.028 (2)	0.053 (3)	0.025 (2)	0.0003 (19)	−0.0093 (17)	−0.0110 (19)
C10	0.0200 (17)	0.0176 (18)	0.026 (2)	−0.0054 (14)	−0.0064 (15)	0.0046 (14)
C11	0.0220 (18)	0.026 (2)	0.0196 (18)	−0.0063 (15)	−0.0036 (15)	0.0001 (15)
C12	0.0253 (19)	0.023 (2)	0.026 (2)	−0.0094 (15)	−0.0108 (16)	0.0056 (15)
C13	0.0176 (17)	0.0210 (19)	0.031 (2)	−0.0011 (14)	−0.0054 (15)	−0.0033 (15)
C14	0.0189 (18)	0.030 (2)	0.0243 (19)	−0.0042 (15)	0.0006 (15)	−0.0064 (16)
C15	0.0227 (18)	0.030 (2)	0.0179 (18)	−0.0043 (16)	−0.0046 (15)	0.0036 (15)
C16	0.0208 (17)	0.0196 (19)	0.027 (2)	−0.0033 (15)	−0.0076 (15)	0.0042 (15)
C17	0.0143 (16)	0.0236 (19)	0.0185 (18)	−0.0059 (14)	−0.0021 (13)	0.0023 (14)
C18	0.0196 (17)	0.0153 (18)	0.028 (2)	−0.0020 (14)	−0.0057 (15)	0.0013 (14)
C19	0.0190 (17)	0.0210 (19)	0.0215 (19)	−0.0072 (14)	−0.0043 (14)	0.0018 (14)
C20	0.028 (2)	0.0214 (19)	0.025 (2)	0.0013 (16)	−0.0037 (16)	0.0007 (16)
C21	0.0236 (18)	0.0176 (18)	0.0222 (19)	−0.0061 (15)	−0.0059 (14)	0.0014 (14)
C22	0.031 (2)	0.0207 (19)	0.0220 (19)	−0.0058 (16)	−0.0067 (16)	−0.0025 (15)

### Geometric parameters (Å, °)

**Table:**

N1—C10	1.276 (5)	C11—H11A	0.9900
N1—C11	1.452 (5)	C11—H11B	0.9900
N2—C21	1.276 (5)	C12—C13	1.389 (5)
N2—C22	1.446 (5)	C12—C17	1.401 (5)
C1—C2	1.380 (5)	C12—H12	0.9500
C1—C6	1.403 (5)	C13—C14	1.380 (5)
C1—H1	0.9500	C13—H13	0.9500
C2—C3	1.384 (5)	C14—C15	1.388 (5)
C2—H2	0.9500	C14—H14	0.9500
C3—C4	1.375 (5)	C15—C16	1.380 (5)
C3—H3	0.9500	C15—H15	0.9500
C4—C5	1.382 (5)	C16—C17	1.401 (5)
C4—H4	0.9500	C16—H16	0.9500
C5—C6	1.401 (5)	C17—C18	1.464 (5)
C5—H5	0.9500	C18—C19	1.349 (5)
C6—C7	1.465 (5)	C18—H18	0.9500
C7—C8	1.354 (5)	C19—C21	1.462 (5)
C7—H7	0.9500	C19—C20	1.502 (5)
C8—C10	1.463 (5)	C20—H20A	0.9800
C8—C9	1.497 (5)	C20—H20B	0.9800
C9—H9A	0.9800	C20—H20C	0.9800
C9—H9B	0.9800	C21—H21	0.9500
C9—H9C	0.9800	C22—C22^ii^	1.534 (7)
C10—H10	0.9500	C22—H22A	0.9900
C11—C11^i^	1.536 (7)	C22—H22B	0.9900
			
C10—N1—C11	117.7 (3)	H11A—C11—H11B	108.2
C21—N2—C22	117.0 (3)	C13—C12—C17	120.8 (3)
C2—C1—C6	120.5 (3)	C13—C12—H12	119.6
C2—C1—H1	119.8	C17—C12—H12	119.6
C6—C1—H1	119.8	C14—C13—C12	120.6 (3)
C1—C2—C3	120.7 (3)	C14—C13—H13	119.7
C1—C2—H2	119.7	C12—C13—H13	119.7
C3—C2—H2	119.7	C13—C14—C15	119.6 (3)
C4—C3—C2	119.9 (3)	C13—C14—H14	120.2
C4—C3—H3	120.0	C15—C14—H14	120.2
C2—C3—H3	120.0	C16—C15—C14	119.8 (3)
C3—C4—C5	119.8 (3)	C16—C15—H15	120.1
C3—C4—H4	120.1	C14—C15—H15	120.1
C5—C4—H4	120.1	C15—C16—C17	121.8 (3)
C4—C5—C6	121.4 (3)	C15—C16—H16	119.1
C4—C5—H5	119.3	C17—C16—H16	119.1
C6—C5—H5	119.3	C12—C17—C16	117.3 (3)
C5—C6—C1	117.6 (3)	C12—C17—C18	125.6 (3)
C5—C6—C7	116.9 (3)	C16—C17—C18	117.1 (3)
C1—C6—C7	125.5 (3)	C19—C18—C17	132.0 (3)
C8—C7—C6	131.0 (3)	C19—C18—H18	114.0
C8—C7—H7	114.5	C17—C18—H18	114.0
C6—C7—H7	114.5	C18—C19—C21	116.0 (3)
C7—C8—C10	116.5 (3)	C18—C19—C20	126.8 (3)
C7—C8—C9	126.6 (3)	C21—C19—C20	117.2 (3)
C10—C8—C9	116.9 (3)	C19—C20—H20A	109.5
C8—C9—H9A	109.5	C19—C20—H20B	109.5
C8—C9—H9B	109.5	H20A—C20—H20B	109.5
H9A—C9—H9B	109.5	C19—C20—H20C	109.5
C8—C9—H9C	109.5	H20A—C20—H20C	109.5
H9A—C9—H9C	109.5	H20B—C20—H20C	109.5
H9B—C9—H9C	109.5	N2—C21—C19	123.5 (3)
N1—C10—C8	122.7 (3)	N2—C21—H21	118.2
N1—C10—H10	118.6	C19—C21—H21	118.2
C8—C10—H10	118.6	N2—C22—C22^ii^	110.4 (4)
N1—C11—C11^i^	109.5 (3)	N2—C22—H22A	109.6
N1—C11—H11A	109.8	C22^ii^—C22—H22A	109.6
C11^i^—C11—H11A	109.8	N2—C22—H22B	109.6
N1—C11—H11B	109.8	C22^ii^—C22—H22B	109.6
C11^i^—C11—H11B	109.8	H22A—C22—H22B	108.1
			
C6—C1—C2—C3	0.9 (6)	C17—C12—C13—C14	0.8 (5)
C1—C2—C3—C4	0.4 (6)	C12—C13—C14—C15	0.7 (5)
C2—C3—C4—C5	0.0 (5)	C13—C14—C15—C16	−1.2 (5)
C3—C4—C5—C6	−1.6 (5)	C14—C15—C16—C17	0.2 (5)
C4—C5—C6—C1	2.8 (5)	C13—C12—C17—C16	−1.8 (5)
C4—C5—C6—C7	−176.2 (3)	C13—C12—C17—C18	−179.5 (3)
C2—C1—C6—C5	−2.4 (5)	C15—C16—C17—C12	1.3 (5)
C2—C1—C6—C7	176.5 (3)	C15—C16—C17—C18	179.2 (3)
C5—C6—C7—C8	165.1 (4)	C12—C17—C18—C19	−12.3 (6)
C1—C6—C7—C8	−13.8 (6)	C16—C17—C18—C19	169.9 (4)
C6—C7—C8—C10	−177.0 (3)	C17—C18—C19—C21	178.9 (3)
C6—C7—C8—C9	1.1 (6)	C17—C18—C19—C20	−0.6 (6)
C11—N1—C10—C8	−178.6 (3)	C22—N2—C21—C19	−179.8 (3)
C7—C8—C10—N1	178.6 (3)	C18—C19—C21—N2	−178.9 (3)
C9—C8—C10—N1	0.2 (5)	C20—C19—C21—N2	0.6 (5)
C10—N1—C11—C11^i^	131.7 (4)	C21—N2—C22—C22^ii^	−124.0 (4)

Symmetry codes: (i) −*x*+1, −*y*+2, −*z*; (ii) −*x*+1, −*y*+1, −*z*.
